# Treatment patterns and outcomes in light chain amyloidosis: An institutional registry of amyloidosis report in Argentina

**DOI:** 10.1371/journal.pone.0274578

**Published:** 2022-10-27

**Authors:** Maria Lourdes Posadas-Martinez, María Adela Aguirre, Erika Brulc, Maria Soledad Saez, Patricia Sorroche, Gerardo Machnicki, Mariana Fernandez, Elsa Mercedes Nucifora

**Affiliations:** 1 Internal Medicine Department, Internal Medicine Research Unit, CONICET, Hospital Italiano de Buenos Aires, Buenos Aires, Argentina; 2 Internal Medicine Department, Hospital Italiano de Buenos Aires, Buenos Aires, Argentina; 3 Internal Medicine Department, Hematology Service, Hospital Italiano de Buenos Aires, Buenos Aires, Argentina; 4 Internal Medicine Department, Biochemestry Service, Hospital Italiano de Buenos Aires, Buenos Aires, Argentina; 5 Janssen-Cilag Farmacêutica Ltda, Buenos Aires, Argentina; 6 Janssen-Cilag S A, Madrid, Spain; 7 Hematology Service, Internal Medicine Department, Hospital Italiano de Buenos Aires, Buenos Aires, Argentina; The University of Texas MD Anderson Cancer Center, UNITED STATES

## Abstract

Light chain (AL) amyloidosis is a form of systemic amyloidosis, causing organ dysfunction, mainly affecting the heart and kidney. Patient-tailored and risk-adapted decision making is critical in AL amyloidosis management. There is limited real-world evidence data from Argentina and Latin America regarding the treatment approaches for AL amyloidosis. This retrospective cohort study aimed to describe the treatment patterns and outcomes in adult patients (>18 years) diagnosed with AL amyloidosis at the Hospital Italiano in Buenos Aires, Argentina, using a 10-yearfollow-up data (June 1, 2010 to May 31, 2019) from the institutional registry of amyloidosis (IRA). The study population had a mean age of 63 years and 54.4% weremale. Heart and kidney were the most frequently affected organs. Of the 90 eligible patients included in the study, 70underwent treatment. Bortezomib-based regimen was the preferred first-line treatment (75.7% patients). Overall,54.4% of the patients presented a deep response (complete or very good partial response). Median overall survival (OS) was 5years, the 1-year OS and progression free survival rates were 80% (95% confidence interval [CI]: 68–87) and 80% (95%CI 68–87)), respectively. This study provides vital real-world evidence for the long-term treatment patterns and survival in a large cohort of AL amyloidosis patients in Argentina.

## Introduction

Immunoglobulin light chain (AL) amyloidosis is the most common form of systemic amyloidosis [1,2]. In AL amyloidosis, amyloid fibrils are formed due to the misfolding of the amyloidogenic light chains, which are produced by the clonal population of plasma cells in the bone marrow [3]. The amyloid fibrils accumulate in various tissues and organs, eventually causing malfunction and organ failure. AL amyloidosis is a rare condition, with an estimated prevalence of 51.27 per million individuals worldwide [2]. The prevalence is on the rise in the US, with a reported estimative of 50.1 cases per million people [4]. In the Argentinian population, an estimative of 11 cases per 1,000,000 person-years have been reported in a prospective cohort study [5]. The prognosis of AL amyloidosis is poor, with a median survival of approximately 13 months in untreated patients [6]. The heart and kidney are the most commonly affected organs (70%cases) in AL amyloidosis, followed by liver and soft tissue (17%), autonomic nervous system (15%) and gastrointestinal tract (10%) [7].

Treatment of AL amyloidosis aims to promptly reduce the supply of newly formed amyloidogenic monoclonal light chains by suppressing the aberrant amyloidogenic clone, consequently reducing the formation of fibrils which allows gradual organ function recovery and improved survival [8]. Currently, there are limited drugs exclusively approved by the health authorities for AL amyloidosis [9,10]. The practice guidelines, however, offer a wide range of therapeutic modalities [11]. The therapeutic landscape for AL amyloidosis has evolved over the years, since the introduction of melphalan, autologous stem cell transplant (ASCT) and conventional chemotherapies. In this scenario, novel strategies like bortezomib (a proteasome inhibitor), lenalidomide-based regimens, and most recently, daratumumab–a human IgG1κ monoclonal antibody that targets the CD38 surface antigen on the plasma cells–have become important therapeutic options. Also, the Food and Drug Administration (FDA)—the US health regulatory agency—recently approved the first treatment combination specifically indicated for AL amyloidosis treatment (daratumumab plus cyclophosphamide, bortezomib and dexamethasone [CyBorD]) [12].

Since patients with AL amyloidosis are typically prone to treatment-related toxicities, an individualized treatment approach is essential while managing these patients. Initial treatment with high dose melphalan and ASCT showed improvements in the quality of life(QoL) and survival outcomes of AL amyloidosis patients [13]. However, this treatment is not recommended for all patients due to the cardiac involvement, to the ASCT ineligibility and/or to the non-tolerability to high doses of melphalan. Cardiac involvement and the extent of cardiac involvement are the major determinants of poor outcomes, but other factors such as age, systolic blood pressure, creatinine levels, Eastern Cooperative Oncology Group (ECOG) performance status [14], presence of large pleural effusions and dependency on oxygen [15,16] have also been identified as prognostic factors related to poor outcomes. In such patients, conventional chemotherapy or bortezomib-based therapy is the preferred option [17]. Treatment with bortezomib, melphalan and dexamethasone demonstrates higher rates of complete response (CR) than melphalan and dexamethasone (42% versus 19%) in the AL amyloidosis patients [18]. Bortezomib-alkylator-steroid combination is preferred for patients with advanced disease—cardiac involvement, renal impairment, severe hypoalbuminemia, fluid retention -as they need a more rapid response [11]. Bortezomib is very effective but still has a significant toxicity profile for advanced cardiac stage patients. Daratumumab demonstrates a significant rapid and deep hematological response in patients with newly diagnosed AL amyloidosis along with an acceptable safety profile [12,17], especially in advanced cardiac stage (IIIa, IIIb) [12].

A risk-adapted and response-tailored approach, accounting for the patient characteristics, organ involvement and cardiac biomarkers is critical in the management of AL amyloidosis. The description of treatment patterns for AL amyloidosis patients and the assessment of survival and treatment outcomes in a real-world clinical setting are necessary to identify the trends and gaps in the standard of care, assisting the key stakeholders to make informed healthcare decisions. Given the paucity of epidemiological data on AL amyloidosis in Argentina, the objective of this study was to evaluate the treatment patterns and outcomes for AL amyloidosis in this region. We used data from the institutional registry of amyloidosis (IRA)–a population-based registry system of amyloidosis patients at the Hospital Italiano in Buenos Aires (HIBA), Argentina.

## Methods

### Study design and participants

This was a retrospective cohort study of adult patients (aged≥18 years) diagnosed with AL amyloidosis and treated at HIBA or referred to it from other centers. Patients’ data available in IRA database between June 01, 2010 and May 31, 2019were analyzed. Patients diagnosed with other forms of amyloidosis (amyloid A [AA], transthyretin-related hereditary [TTR] amyloidosis, localized amyloidosis, or non-typification of the protein), and those without a signed informed consent form were excluded from the analyses. The study was approved by the local institutional review board (IRB)–N5286.

### Data source

HIBA has been retrospectively collecting amyloidosis case data in the IRA single center registry since 2010.The IRA is registered in ClinicalTrials.gov (NCT01347047) and is approved by the IRB(CEPI 1635).Diagnosis of AL amyloidosis was confirmed by the presence of amyloid fibrils in the tissue, in addition to the demonstration of a monoclonal plasma cell proliferative disorder and the presence of amyloid-related systemic syndrome [19]. The registry includes data for baseline demographics, medical history, comorbidities, physical examinations, laboratory, and imaging exams. Treatments were prescribed by the physician of the amyloidosis team in HIBA, according to the hospital protocol. The criterion for heart transplantation was non-reversible heart failure with poor willingness to optimal treatment, with limiting symptoms, and with a poor short-term prognosis. Additionally, the registry also captures data for the overall survival (OS: time from diagnosis to death), progression free survival (PFS: time from diagnosis to disease progression or death) and the overall hematological response (as per response criteria by the International Myeloma Working Group [20], complete response [CR], very good partial response [VGPR], partial response [PR], no response [NR]). A full-time research fellow screened all the patients at the initial diagnosis and updated the database during the follow-up visits (frequency as per the physician´s discretion). The data were collected in a standardized electronic case report form and stored as per the requisite quality standards.

### Outcome measures

The inclusion criterion of the study was the confirmation of AL amyloidosis diagnosis. The primary outcome was the description of overall treatment patterns for AL amyloidosis over 10-year period. The secondary outcomes included: OS and PFS in the overall population, OS in the patient subgroups stratified by the ASCT status, hematological response rate in the intention-to-treat population (ITT, defined as patients with available best response to any line of therapy), number of treatment lines and number of cycles per line of treatment received. Survival analysis were performed in a subset of treated patients. Additionally, baseline demographics and clinical characteristics were assessed in all eligible patients.

### Statistical analysis

The statistical analyses were performed with Stata version 13.0. The continuous variables were presented as mean and standard deviation (SD)or median and interquartile range (IQR), and the categorical variables were presented as absolute frequencies and percentages. The OS and PFS were estimated by the Kaplan–Meier (KM) method. The log rank test was used to compare the OS between the ASCT versus non-ASCT subgroup. The cox regression analysis was performed, and the median survival time and 95%confidence interval (CI) were calculated. Patients were censored at end of follow up, last data entry, last recorded visit or administrative censoring at 01 May 2019.

## Results

### Participants

**Fig 1:**
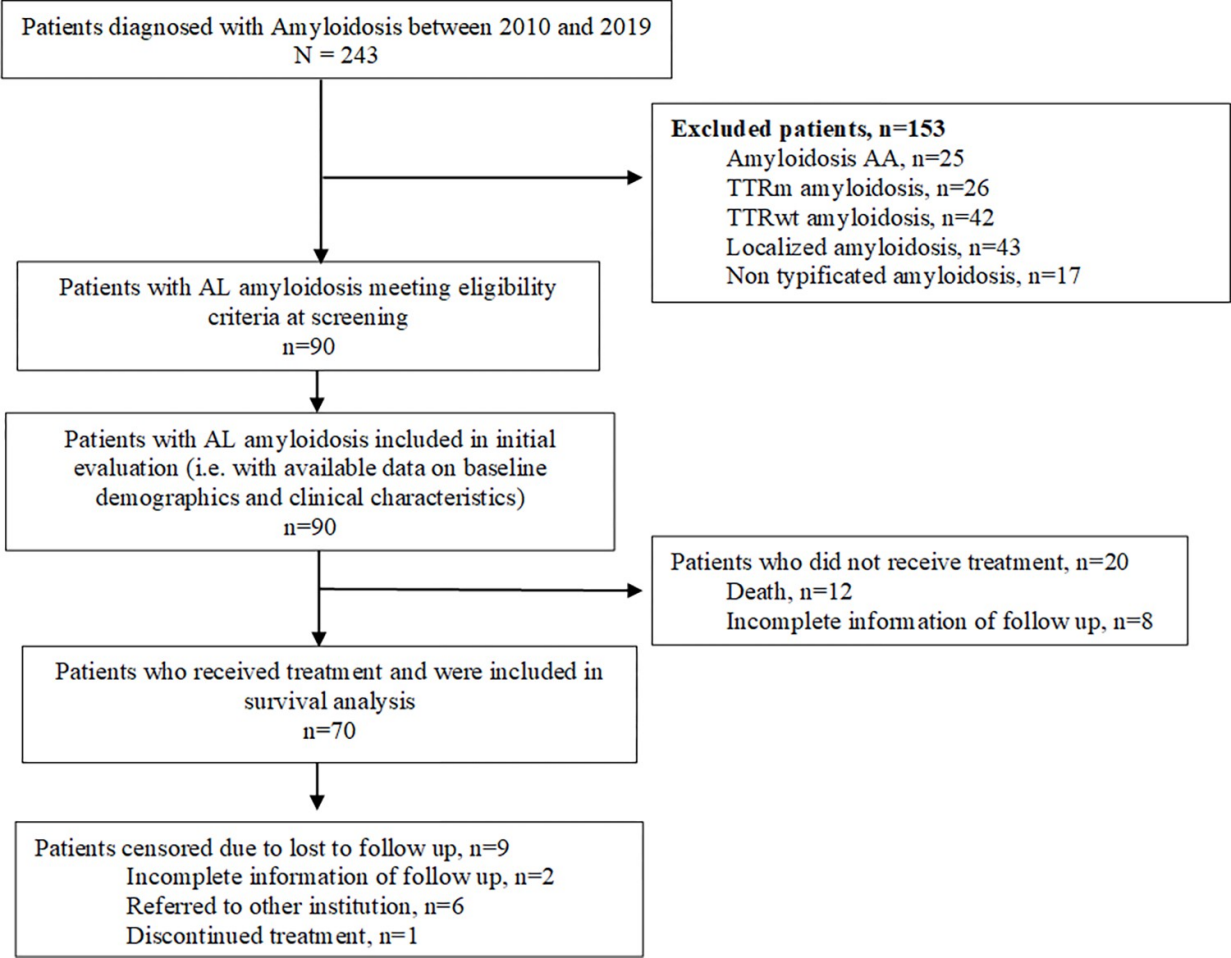
Patient disposition flow chart. AA amyloidosis: Serum amyloid A amyloidosis; AL amyloidosis: Light chain amyloidosis; TTRm amyloidosis: Mutational transthyretin amyloidosis; TTRwt: Wild type transthyretin amyloidosis.

A total of 243 patients with amyloidosis was identified from the IRA database and assessed for study eligibility. Among these patients, 37.0% patients (n = 90/243) met the study eligibility criteria. Overall, 62.9% patients (n = 153/243) were excluded from the study due to diagnosis of other forms of amyloidosis (AA [n = 25/153], mutational transthyretin [TTRm] amyloidosis [n = 26/153], wildtype transthyretin [TTRwt] amyloidosis [n = 42/153], localized amyloidosis [n = 43/153] and non-typified amyloidosis [n = 17/153]).Data were extracted for all the 90 eligible patients. Among them, 70patientsreceived treatment for AL amyloidosis. Twenty patients were excluded from the analysis due to incomplete treatment-related information in the IRA: 12 patients died due to disease severity before treatment initiation, 8 patients had incomplete information as they were referred from other hospitals. Patient disposition is presented in Fig 1.

### Baseline characteristics

The patient was included in the database at the time of diagnosis; thus, the baseline data refer to the closest time to the diagnosis Baseline characteristics were assessed for all the 90 eligible patients. The mean age at diagnosis was 63 years at diagnosis, and 54.4% were men (n = 49/90). Overall, the study population had a mean of 2 organs involved with amyloid (Table 1). The most affected organs were heart (72.2% [n = 65/90]) and kidney (68.9% [n = 62/90]). The majority of patients had an ECOG grade of 1 or 2 (n = 71/90). The mean duration from symptoms to diagnosis was 6months [IQR1-14]. The mean estimated glomerular filtration rate (eGFR) was 60 mL/min/1.73m^2^ and median creatinine was 1.1 mg/dL. The median alkaline phosphatase was 101 IU/L, while the median NT-ProBNP was 3,771 pg/mL (Table 1).

**Table 1:** Baseline characteristics of AL amyloidosis patients.

	N = 90
**Age at diagnosis, years, mean± SD**	63 ± 13
**Gender, n (%)**	
Men	49 (54.4)
Women	41 (45.6)
**Charlson score, mean ± SD**	2 ± 2
**ECOG performance status ^1^**	
ECGO score, mean ± SD	2 ± 1
ECOG grades, n (%)	
0–1	35 (38.9)
2	37 (41.1)
≥3	18 (20)
**Time from symptoms until diagnosis, months, mean ± SD**	1.5 ± 2.8^a^
**Involved free light chain type**	
Lambda, n (%)	61 (71.7)^b^
Kappa, n (%)	24 (28.2)^b^
Lambda, mg/L, median (interquartile range)	143 (70–315)^c^
Kappa, mg/L, median interquartile range)	161 (68–448)^d^
Kappa/Lambda ratio, (mean± SD)	15.34 ± 83 ^e^
dFLC,median (interquartile range)	110 (37–200) ^f^
**Serum beta-2 microglobulin, mg/dL, median (interquartile range)**	0.5 (0.3–1.3)^g^
**Bone marrow ≥10% plasma cells, n (%)**	49 (54.4)
**Monoclonal Spike proteinogram, n (%)**	39 (41.1)
**Organ involvement, n (%) ^2^**	
Cardiac	65 (72.2)
NYHAclass^h^	
I	8 (14%)
II	23 (41%)
III	19 (34%)
IV	6 (11%)
Renal	62 (68.9)
Peripheral neuropathy	32 (35.6)
Liver	15 (16.5%)
Gastrointestinal	33 (36.7)
Soft tissue	11 (12%)
Numbers of organs involved (except soft tissue), mean ± SD	2 ± 1
**Biomarkers, mean ± SD**	
eGFR, mL/min/1.73 m^2^	60 ± 51^i^
Creatinine, mg/dL	1.1 (0.8–2.2)^j^
Proteinuria>150, mg/24hs (n 72)	51(71%)
Alkaline phosphatase, IU/L	101 (66–151)^k^
Troponin (n 52), ng/mL, median (interquartile range)	56 (10–154)
NT-Pro BNP, pg/mL, median (interquartile range)	3771 (1349–10607)^l^
NT-Pro BNP > 8500 pg/mL	21 (32%)^l^

dFLC, difference between involved and uninvolved free light chain; eGFR: Estimated glomerular filtration rate; NT-Pro-BNP: N-Terminal-Pro B-type natriuretic peptide; N: Number; SD: Standard Deviation.

^1^ECOG criteria(14): Grade 0, fully active, able to carry on all pre-disease performance without restriction; grade 1, Restricted in physically strenuous activity but ambulatory and able to carry out work of a light or sedentary nature, e.g., light housework, office work; grade 2, Ambulatory and capable of all selfcare but unable to carry out any work activities. Up and about more than 50% of waking hours; grade 3, Capable of only limited selfcare, confined to bed or chair more than 50% of waking hours; grade 4, Completely disabled. Cannot carry on any selfcare. Totally confined to bed or chair; grade 5, dead.

^2^Organ involvement was defined according to Gertz et al [21] and Gertz y Merlini,Amyloid [22].

^a^Analyzed in 66 patients

^b^Analyzed in 85 patients

^c^Analyzed in 61 patients

^d^Analyzed in 24 patients

^e^Analyzed in 82 patients

^f^Analyzed in 85 patients

^g^Analyzed in 52 patients

^h^Analyzed in 56 patients

^i^Analyzed in 32 patients

^j^Analyzed in 77 patients

^k^Analyzed in 84 patients

^l^Analyzed in 64 patients.

### Treatment pattern

A total of 78.5% patients (n = 70/90) received treatment for AL amyloidosis and were followed up for a median duration of 15months (IQR5-45). Among the 70 patients receiving chemotherapy, 24.2% (n = 16/70) received chemotherapy followed by ASCT, 11.4% (n = 8/70) received chemotherapy after heart transplant, while one patient each (1.5%) received heart and kidney transplant, respectively, post-chemotherapy.

The most frequently prescribed first-line chemotherapy was a bortezomib-based regimen (75.7% patients [n = 53/70]), with a median of 4cycles (IQR: 3–5). Overall, 38.8% patients (n = 26/67) received second-line treatment, with a median of 4 cycles (IR: 2–6). Lenalidomide/dexamethasone was the most frequently second-line combination prescribed (42.3% [n = 11/26]). Few patients required third-line therapy (4.5% [n = 3/67]), which included bortezomib-based regimens (median cycles: 6 [IQR: 1–6]). Only one patient was administered a fourth-line treatment (combination of daratumumab-dexamethasone; median cycles: 6) (Table 2).

**Table 2:** Regimens used for AL amyloidosis patients per line of treatment.

Patients who received chemotherapy	N = 70
**Front line treatment**	**n = 70**
***Regimens*, *n (%)***	
Bortezomib-based regimens	53 (75.7)
Thalidomide-Dexamethasone/Cyclophosphamide	8 (11.4)
Melphalan-Dexamethasone	6 (8.6)
Rituximab -Cyclophosphamide vincristine Sulphate and Prednisone*	1 (1.4)
Lenalidomide-Dexamethasone	1 (1.4)
Rituximab Prednisone*	1 (1.4)
**Second line treatment (n = 26) (n, %)**	**n = 26**
***Regimens*, *n (%)***	
Lenalidomide-Dexamethasone	11 (42.3)
Daratumumab- Dexamethasone	5 (19.2)
Melphalan-Dexamethasone	5(19.3)
Bortezomib-based regimens	2 (7.7)
Thalidomide-Dexamethasone/Cyclophosphamide	1 (3.8)
Lenalidomide-bortezomib -dexamethasone (RVD)	1 (3.8)
Rituximab bendamustine	1 (3.8)
**Third line treatment**	**n = 3**
***Regimens*, *n (%)***	
Bortezomib-based regimens	3 (100)
**Fourth line treatment**	**n = 1**
***Regimen*, *n (%)***	
Daratumumab-dexamethasone	1 (1.5)

*IgM patient with Non-Hodgkin Lymphoma has been treated with Rituximab -Cyclophosphamide vincristine Sulphate and Prednisoneafter heart transplantation.

### Hematologic response

Hematologic response was evaluated in 82.8% patients on chemotherapy (n = 58/70), with majority receiving only one line of therapy (n = 43/70). In 15.7% of patients (n = 11/70), the response was not assessed (death [n = 2], patient’s decision to discontinue treatment[n = 1], insufficient follow-up time[n = 2], referral to other institutions[n = 6]).The best hematologic response in any line of therapy(ITT population) were CR in 47.1% patients (n = 33/70), VGPR in 10% patients (n = 7/70), PR in 4.3% patients (3/70) and NR in 22.9% patients (n = 16/70). The relapse rate was 22% (n = 9, 95% CI: 11–38).

### Survival

**Fig 2:**
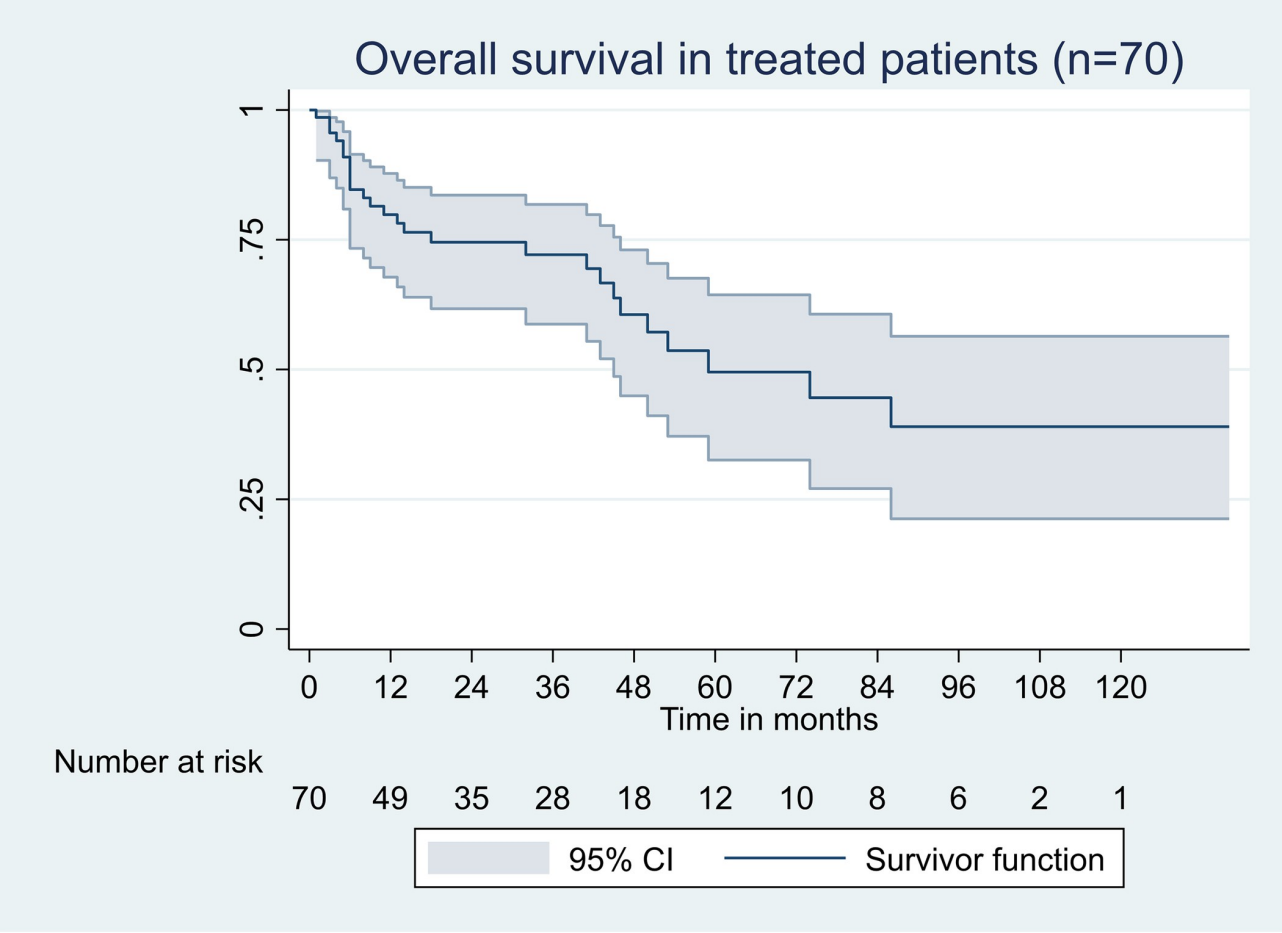
Overall survival in treated patients.

**Fig 3:**
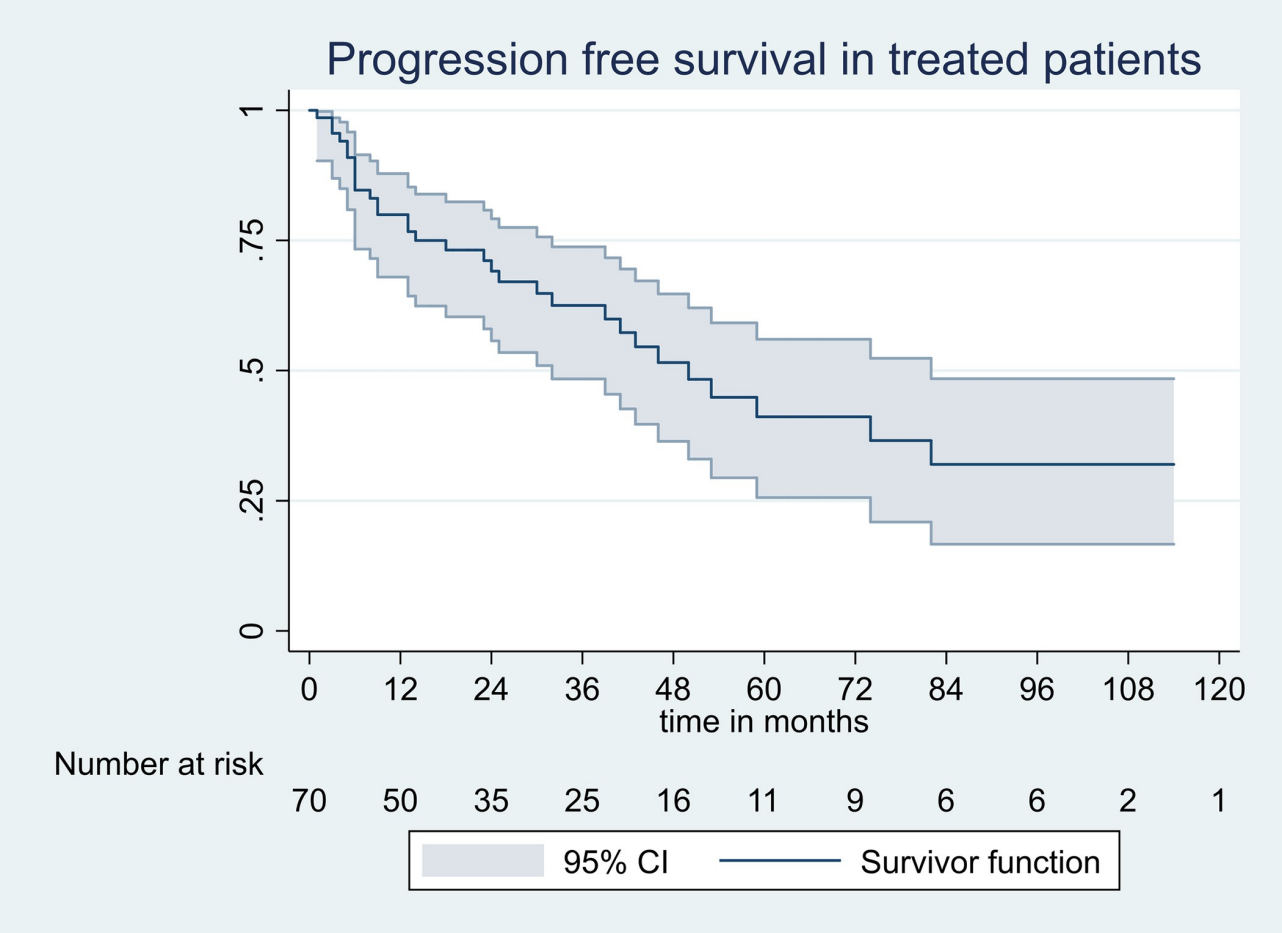
Progression free survival. CI, confidence intervals.

A total of 24 patients died within 12 months, of which 12 died prior to treatment initiation as a result of advanced disease and irreversible organ dysfunction (survival of untreated patients at 12 months:70%;95% CI: 59–80). Nine patients among those who died after receiving a treatment achieved a deep hematological response. Majority of deaths were related to cardiac events(n = 19). Overall, the median OS in treated patients was 5 years. The OS rate at 1 year was 80% (95% CI: 68–87), at 5 years was 50% (95% CI: 32–64) and at 10 years was 40% (95% CI: 21–56) (Fig 2). The PFS rates was80% (95%CI 68–87) at first year, 41% (95%CI: 26–56) at five years and 32% (95%CI: 16–48) at 10 years (Fig 3). The 1 and 5-year OS rates in the two cohorts (with ASCT versus without ASCT, log rank = 0.16) were100% versus74% and 70% versus 43%, respectively. There was no treatment related mortality at day 100 after ASCT.

## Discussion

In the recent years, the development of novel therapeutic options, along with a better understanding of the supportive care, has improved the prognosis of patients with AL amyloidosis [23]. However, the early mortality rate related to the disease is still high, mainly due to the delay in diagnosis [24]. Patients presenting with advanced cardiac stage have a median survival of less than 6 months, if left untreated [24,25]. Patients that receive treatment have a better outcomes [23,26,27]. Treating AL amyloidosis patients is challenging due to the reduced functional organ reserves of their organs, as a result of amyloid deposits, which magnifies the increases susceptibility to treatment toxicity and limits the administration of an effective therapeutic dose. full dose administration. Additionally, a multidisciplinary approach and expert supportive treatment are essential for the management of a systemic rare disease as AL amyloidosis. Effective treatment administration and a patient-tailored approach Understanding which are the most effective treatment strategies and adapt them to a patient-tailored approach is are of paramount importance. To our knowledge, this is the first study that investigates the treatment patterns and outcomes in systemic AL amyloidosis patients in Argentina. Here, we used data from the IRA over a ten-year period (from 2010 to 2019). The study population had a mean age of 63 years, with comparatively more men than women (54.4% men), consistent with other studies on AL amyloidosis [16,28,29]. In this population, heart and kidney were the most affected organs, and the majority of patients had monoclonal light chain of lambda type. Overall, 13% patients died before treatment, probably due to a delay in diagnosis [27].

Most of the patients were eligible to receive chemotherapy as the first-line therapy and only eight patients were eligible for upfront heart transplantation to prevent death and improve tolerance to upcoming chemotherapy. The individualized treatment was decided upon the hospital’s amyloidosis team, considering various factors like organ function, performance status, age, patient’s preferences and availability of social insurance, which was in line with the previous long-term studies on patients with AL amyloidosis. Of the 70 patients in whom upfront treatment with chemotherapy could be ascertained, bortezomib-based regimens were the most used, in 75.7% patients, followed by thalidomide-dexamethasone/cyclophosphamide (11.4%) and melphalan-dexamethasone (8.6%) combinations. The treatment trends observed in this Argentinian population were in line with the global standards for AL amyloidosis(8,11). Only one in five patients receiving chemotherapy required ASCT, which is known to prolong survival and improve prognosis [23]. Overall, about 55.7% (n = 39/70) patients receiving chemotherapy achieved a deep hematological response (CR or VGPR), with CR present in 47.1% patients (n = 33/70) and VGPR in10% patients (n = 7/70). These findings corroborate the study by Palladini et al, in which 60% of patients achieved either a CR or VGPR(23% achieved CR) [30]. The number of treatment cycles varied depending upon the achievement of response, intention to perform an ASCT and incidence of adverse events. The most commonly used second-line treatment regimen in this study was lenalidomide-dexamethasone (n = 11) and daratumumab (n = 5), which was also in line with previous studies [31,32]. The recent phase III Andromeda study showed that daratumumab plus CyBorD was superior to CyBorD alone in achieving CR in AL amyloidosis patients, although daratumumab is not yet approved in Argentina for AL amyloidosis patients [12,29,33]. The development of novel monoclonal antibodies that directly act on amyloid deposits, like CAEL-101, is very promising and results are eagerly awaited to determine their role in the management of these patients.]. In AL amyloidosis, mortality is multifactorial and depends on the performance status, type and severity of organ involvement, and clonal behavior [25,31,32]. In this study, the median OS was five years, while death was recorded in about 37.1% patients (n = 26/70) who received treatment. Notably, most of the deaths occurred in patients with cardiac involvement (73.1%; n = 19/26 deaths). Among those who died during the study period, only nine patients achieved a deep hematological response. Most of these patients had a lack of response to treatment, however this could be because these patients did not survive long enough to demonstrate an optimal treatment effect. This is consistent with reports indicating that cardiac involvement is by far the main prognostic determinant in AL amyloidosis. The mortality predictors in this study were similar to a10-year study conducted in amyloidosis Center at the Boston University Medical Center [24]. The 1-yearOS rate was similar to other published studies and can be attributed to proper selection of patients, treatment regimens and transdisciplinary approaches of the center [28,34,35].

This study provides substantial real-world data from a large amyloidosis treatment setting in Argentina over a 10-year period. The AL amyloidosis treatment pattern in Argentina shows a progressive trend to include the use of traditional drugs like thalidomide as an upfront treatment, with a progressive shift from the use of traditional alkylating agents as upfront treatment to the use of novel drugs like bortezomib [36]. However, there is a compelling need to adopt new technologies at the study center, similar to other developed countries. Most of the patients in the study were followed comprehensively until the end of study, however, for some patients, their health insurance did not approve certain diagnostic tests. While only a few patients had an early diagnosis, most of the patients were assessed only after a multi-organ involvement. Adequate treatment could not be ascertained in patients with late diagnosis, highlighting the importance of early diagnosis and treatment in patients with AL amyloidosis [37].

We do acknowledge certain limitations in this study. Since it is a secondary database study based on patient registry, the study could only evaluate variables that were available in the registry. Nonetheless, the variables in the registry are absolute and widely available, which should improve its internal validity. In addition, data were collected using standardized electronic forms, double checking was performed during data collection, and data were assessed to confirm consistency of information.

Finally, since we evaluated a 10-year database period, several changes in guidelines and treatment choices occurred, which may have impacted the data analysis. Besides, the lack of availability of minimal residual disease (MRD)assessment is also a limitation, as this is a very important factor in terms of prognosis and disease outcome. In future studies, this data may be useful to better understand patients’ level of response to the drugs used in clinical practice.

The treatment of AL Amyloidosis in Argentina evolved over the years as different drugs were approved. There are currently no specifically approved treatments options for AL amyloidosis, and because of that, multiple myeloma treatment regimens are extrapolated.

The main strength of this study is that IRA cases were prospectively collected in a standardized data collection form and followed-up to ensure a high-quality data by minimizing the loss of follow-up. As an observational study, the data depicts the medical practice for patients with amyloidosis at a teaching tertiary care hospital. The IRA registry has important clinical history of AL amyloidosis patients, including diagnosis, treatment, and prognosis of these individuals. These data could be used to improve clinical outcomes and quality of life for patients. Finally, to the best of our knowledge, no other studies have included such a large number of patients with AL amyloidosis in Latin America [38,39]

## Conclusion

This study highlights the importance of early diagnosis and treatment for patients with AL amyloidosis. It provides a vital real-world evidence for the diagnosis and treatment patterns for patients with AL amyloidosis in the Argentinian population, which would be useful for clinical decision making and help revolutionize the treatment for patients with AL amyloidosis. More real-world evidence data is required to further improve the patient care, and meet the unmet need, in terms of management of AL amyloidosis.

## Supporting information

S1 Fig Overall survival. CI, confidence intervals.
(TIF)

## References

[1] Palladini G, Merlini G. What is new in diagnosis and management of light chain amyloidosis? Blood [Internet]. 2016 Jul 14;128(2):159–68. Available from: https://ashpublications.org/blood/article/128/2/159/35451/What-is-new-in-diagnosis-and-management-of-light. doi: 10.1182/blood-2016-01-629790 27053535

[2] Zhang N, Cherepanov D, Romanus D, Kumar N, Hughes M, Faller D V. Estimating the Global Epidemiology of Amyloid Light-Chain Amyloidosis With an Incidence-to-Prevalence Model. Clin Lymphoma Myeloma Leuk [Internet]. 2019 Oct;19(10):e339. Available from: https://linkinghub.elsevier.com/retrieve/pii/S2152265019319457.

[3] Rosenzweig M, Landau H. Light chain (AL) amyloidosis: update on diagnosis and management. J Hematol Oncol [Internet]. 2011 Dec 18;4(1):47. Available from: https://jhoonline.biomedcentral.com/articles/10.1186/1756-8722-4-47. doi: 10.1186/1756-8722-4-47 22100031 PMC3228694

[4] Quock TP, Yan T, Chang E, Guthrie S, Broder MS. Epidemiology of AL amyloidosis: a real-world study using US claims data. Blood Adv [Internet]. 2018 May 22;2(10):1046–53. Available from: https://ashpublications.org/bloodadvances/article/2/10/1046/15861/Epidemiology-of-AL-amyloidosis-a-realworld-study. doi: 10.1182/bloodadvances.2018016402 29748430 PMC5965052

[5] Aguirre MA, Boietti BR, Nucifora E, Sorroche PB, González Bernaldo de Quirós F, Giunta DH, et al. Incidence rate of amyloidosis in patients from a medical care program in Buenos Aires, Argentina: a prospective cohort. Amyloid [Internet]. 2016 Jul 2;23(3):184–7. Available from: https://www.tandfonline.com/doi/full/10.1080/13506129.2016.1207626. 27470486 10.1080/13506129.2016.1207626

[6] Kyle R., Gertz M. Primary systemic amyloidosis: clinical and laboratory features in 474 cases. Semin Hematol. 1995;32(1):45–9. 7878478

[7] Palladini G, Milani P, Merlini G. Novel strategies for the diagnosis and treatment of cardiac amyloidosis. Expert Rev Cardiovasc Ther. 2015;13(11):1195–211. doi: 10.1586/14779072.2015.1093936 26496239

[8] Palladini G, Merlini G. Current treatment of AL amyloidosis. Haematologica [Internet]. 2009 Aug 1;94(8):1044–8. Available from: http://www.haematologica.org/cgi/doi/10.3324/haematol.2009.008912. 19644136 10.3324/haematol.2009.008912 PMC2719026

[9] EMA. European medicines agency. Public summary of opinion on orphan designation; Chimeric fibril-reactive IgG1k monoclonal antibody 11-1F4 for the treatment of AL amyloidosis. 2020; Available from: https://www.ema.europa.eu/en/documents/orphan-designation/eu/3/19/2222-public-summary-opinion-orphan-designation-chimeric-fibril-reactive-igg1k-monoclonal-antibody-11_en.pdf.

[10] AMC. Guidance for Industry AL Amyloidosis—Developing Drugs for Treatment. 2016; Available from: https://www.arci.org/wp-content/uploads/2018/04/Guidance-for-Industry-AL-Amyloidosis-Developing-Drugs-for-Treatment-12_16.pdf.

[11] Wechalekar AD, Gillmore JD, Bird J, Cavenagh J, Hawkins S, Kazmi M, et al. Guidelines on the management of AL amyloidosis. Br J Haematol [Internet]. 2015 Jan;168(2):186–206. Available from: https://onlinelibrary.wiley.com/doi/ doi: 10.1111/bjh.13155 25303672

[12] Kastritis E, Palladini G, Minnema MC, Wechalekar AD, Jaccard A, Lee HC, et al. Daratumumab-Based Treatment for Immunoglobulin Light-Chain Amyloidosis. N Engl J Med [Internet]. 2021 Jul 1;385(1):46–58. Available from: http://www.nejm.org/doi/ doi: 10.1056/NEJMoa2028631 34192431

[13] Sanchorawala V, Skinner M, Quillen K, Finn KT, Doros G, Seldin DC. Long-term outcome of patients with AL amyloidosis treated with high-dose melphalan and stem-cell transplantation. Blood [Internet]. 2007 Nov 15;110(10):3561–3. Available from: https://ashpublications.org/blood/article/110/10/3561/23387/Longterm-outcome-of-patients-with-AL-amyloidosis. doi: 10.1182/blood-2007-07-099481 17673601 PMC2077307

[14] Oken MM, Creech RH, Tormey DC, Horton J, Davis TE, McFadden ET, et al. Toxicity and response criteria of the Eastern Cooperative Oncology Group. Am J Clin Oncol [Internet]. 1982 Dec;5(6):649–55. Available from: http://www.ncbi.nlm.nih.gov/pubmed/7165009. 7165009

[15] National Comprehensive Cancer Network. NCCN clinical practice guidelines in oncology; Systemtic light chain amyloidosis. 2013; Available from: https://www2.tri-kobe.org/nccn/guideline/hematologic/english/amyloidosis.pdf.

[16] Skinner M, Anderson JJ, Simms R, Falk R, Wang M, Libbey CA, et al. Treatment of 100 patients with primary amyloidosis: A randomized trial of melphalan, prednisone, and colchicine versus colchicine only. Am J Med [Internet]. 1996 Mar;100(3):290–8. Available from: https://linkinghub.elsevier.com/retrieve/pii/S0002934397894879. doi: 10.1016/s0002-9343(97)89487-9 8629674

[17] Kastritis E, Leleu X, Arnulf B, Zamagni E, Cibeira MT, Kwok F, et al. Bortezomib, Melphalan, and Dexamethasone for Light-Chain Amyloidosis. J Clin Oncol [Internet]. 2020 Oct 1;38(28):3252–60. Available from: https://ascopubs.org/doi/10.1200/JCO.20.01285. 32730181 10.1200/JCO.20.01285

[18] Palladini G, Milani P, Foli A, Vidus Rosin M, Basset M, Lavatelli F, et al. Melphalan and dexamethasone with or without bortezomib in newly diagnosed AL amyloidosis: a matched case–control study on 174 patients. Leukemia [Internet]. 2014 Dec 25;28(12):2311–6. Available from: http://www.nature.com/articles/leu2014227. doi: 10.1038/leu.2014.227 25059496

[19] Rajkumar SV, Dimopoulos MA, Palumbo A, Blade J, Merlini G, Mateos M-V, et al. International Myeloma Working Group updated criteria for the diagnosis of multiple myeloma. Lancet Oncol [Internet]. 2014 Nov;15(12):e538–48. Available from: https://linkinghub.elsevier.com/retrieve/pii/S1470204514704425. doi: 10.1016/S1470-2045(14)70442-5 25439696

[20] Palladini G, Dispenzieri A, Gertz MA, Kumar S, Wechalekar A, Hawkins PN, et al. New criteria for response to treatment in immunoglobulin light chain amyloidosis based on free light chain measurement and cardiac biomarkers: Impact on survival outcomes. J Clin Oncol. 2012;30(36):4541–9. doi: 10.1200/JCO.2011.37.7614 23091105

[21] Gertz MA, Comenzo R, Falk RH, Fermand JP, Hazenberg BP, Hawkins PN, et al. Definition of organ involvement and treatment response in immunoglobulin light chain amyloidosis (AL): A consensus opinion from the 10th International Symposium on Amyloid and Amyloidosis. Am J Hematol [Internet]. 2005 Aug;79(4):319–28. Available from: https://onlinelibrary.wiley.com/doi/10.1002/ajh.20381. 16044444 10.1002/ajh.20381

[22] Merlini G, Seldin DC, Gertz MA. Amyloidosis: Pathogenesis and New Therapeutic Options. J Clin Oncol [Internet]. 2011 May 10;29(14):1924–33. Available from: http://ascopubs.org/doi/10.1200/JCO.2010.32.2271. 21483018 10.1200/JCO.2010.32.2271 PMC3138545

[23] Ryšavá R. AL amyloidosis: advances in diagnostics and treatment. Nephrol Dial Transplant [Internet]. 2019 Sep 1;34(9):1460–6. Available from: https://academic.oup.com/ndt/article/34/9/1460/5123556. doi: 10.1093/ndt/gfy291 30299492

[24] Tahir UA, Doros G, Kim JS, Connors LH, Seldin DC, Sam F. Predictors of Mortality in Light Chain Cardiac Amyloidosis with Heart Failure. Sci Rep [Internet]. 2019 Dec 12;9(1):8552. Available from: http://www.nature.com/articles/s41598-019-44912-x. doi: 10.1038/s41598-019-44912-x 31189919 PMC6561903

[25] Barrett CD, Dobos K, Liedtke M, Tuzovic M, Haddad F, Kobayashi Y, et al. A Changing Landscape of Mortality for Systemic Light Chain Amyloidosis. JACC Hear Fail [Internet]. 2019 Nov;7(11):958–66. Available from: https://linkinghub.elsevier.com/retrieve/pii/S2213177919305931. doi: 10.1016/j.jchf.2019.07.007 31606365

[26] Sachchithanantham S, Offer M, Venner C, Mahmood SA, Foard D, Rannigan L, et al. Clinical profile and treatment outcome of older (&gt;75 years) patients with systemic AL amyloidosis. Haematologica [Internet]. 2015 Nov 1;100(11):1469–76. Available from: http://www.haematologica.org/cgi/doi/10.3324/haematol.2015.128025. 10.3324/haematol.2015.128025 PMC4825311 26294730

[27] Muchtar E, Gertz MA, Lacy MQ, Go RS, Buadi FK, Dingli D, et al. Ten‐year survivors in AL amyloidosis: characteristics and treatment pattern. Br J Haematol [Internet]. 2019 Dec 12;187(5):588–94. Available from: https://onlinelibrary.wiley.com/doi/ doi: 10.1111/bjh.16096 31298751 PMC6872910

[28] Manwani R, Cohen O, Sharpley F, Mahmood S, Sachchithanantham S, Foard D, et al. A prospective observational study of 915 patients with systemic AL amyloidosis treated with upfront bortezomib. Blood [Internet]. 2019 Dec 19;134(25):2271–80. Available from: https://ashpublications.org/blood/article/134/25/2271/375009/A-prospective-observational-study-of-915-patients. doi: 10.1182/blood.2019000834 31578202

[29] Lecumberri R, Krsnik I, Askari E, Sirvent M, González-Pérez MS, Escalante F, et al. Treatment with daratumumab in patients with relapsed/refractory AL amyloidosis: a multicentric retrospective study and review of the literature. Amyloid [Internet]. 2020 Jul 2;27(3):163–7. Available from: https://www.tandfonline.com/doi/full/ doi: 10.1080/13506129.2020.1730791 32106714

[30] Palladini G, Sachchithanantham S, Milani P, Gillmore J, Foli A, Lachmann H, et al. A European collaborative study of cyclophosphamide, bortezomib, and dexamethasone in upfront treatment of systemic AL amyloidosis. Blood [Internet]. 2015 Jul 30;126(5):612–5. Available from: https://ashpublications.org/blood/article/126/5/612/126432/A-European-collaborative-study-of-cyclophosphamide. doi: 10.1182/blood-2015-01-620302 25987656

[31] Kaufman GP, Schrier SL, Lafayette RA, Arai S, Witteles RM, Liedtke M. Daratumumab yields rapid and deep hematologic responses in patients with heavily pretreated AL amyloidosis. Blood [Internet]. 2017 Aug 17;130(7):900–2. Available from: https://ashpublications.org/blood/article/130/7/900/36992/Daratumumab-yields-rapid-and-deep-hematologic. doi: 10.1182/blood-2017-01-763599 28615223

[32] Sanchorawala V, Sarosiek S, Schulman A, Mistark M, Migre ME, Cruz R, et al. Safety, tolerability, and response rates of daratumumab in relapsed AL amyloidosis: results of a phase 2 study. Blood [Internet]. 2020 Apr 30;135(18):1541–7. Available from: https://ashpublications.org/blood/article/135/18/1541/440750/Safety-tolerability-and-response-rates-of. doi: 10.1182/blood.2019004436 31978210 PMC7193185

[33] Palladini G, Kastritis E, Maurer MS, Zonder J, Minnema MC, Wechalekar AD, et al. Daratumumab plus CyBorD for patients with newly diagnosed AL amyloidosis: safety run-in results of ANDROMEDA. Blood [Internet]. 2020 Jul 2;136(1):71–80. Available from: https://ashpublications.org/blood/article/136/1/71/454281/Daratumumab-plus-CyBorD-for-patients-with-newly. doi: 10.1182/blood.2019004460 32244252 PMC7332897

[34] Sidana S, Dispenzieri A, Murray DL, Go RS, Buadi FK, Lacy MQ, et al. Revisiting complete response in light chain amyloidosis. Leukemia [Internet]. 2020 May 26;34(5):1472–5. Available from: http://www.nature.com/articles/s41375-019-0664-9. doi: 10.1038/s41375-019-0664-9 31772296 PMC8083944

[35] Sloan JM. Lessons in Long Term Survival from AL Amyloidosis. Br J Haematol [Internet]. 2019 Dec 10;187(5):557–8. Available from: https://onlinelibrary.wiley.com/doi/ doi: 10.1111/bjh.16094 31290565

[36] Landau H, Hassoun H, Rosenzweig MA, Maurer M, Liu J, Flombaum C, et al. Bortezomib and dexamethasone consolidation following risk-adapted melphalan and stem cell transplantation for patients with newly diagnosed light-chain amyloidosis. Leukemia [Internet]. 2013 Apr 27;27(4):823–8. Available from: http://www.nature.com/articles/leu2012274. doi: 10.1038/leu.2012.274 23014566

[37] Vaxman I, Gertz M. Recent Advances in the Diagnosis, Risk Stratification, and Management of Systemic Light-Chain Amyloidosis. Acta Haematol [Internet]. 2019;141(2):93–106. Available from: https://www.karger.com/Article/FullText/495455. doi: 10.1159/000495455 30650422

[38] Peña C, Gonzáles JT, López-Vidal H, Donoso J, Contreras C, Vergara CG, et al. AL amyloidosis in the Chilean public health system: a pending debt. Multicenter study of the Chilean Monoclonal Gammopathies Cooperative Group. Artículos Investig. 2019;147:1239–46. doi: 10.4067/s0034-98872019001001239 32186631

[39] Hernández-Reyes J, Galo-Hooker E, Ruiz-Delgado GJ, Ruiz-Argüelles GJ. Systemic immunoglobulin light-chain amyloidosis (AL) in Mexico: A single institution, 30-year experience. Rev Investig Clin. 2012;64(6):604–6. 23593777

