# Supplementary figures and images for: Treatment patterns and outcomes in light chain amyloidosis: An institutional registry of amyloidosis report in Argentina
Source: PLoS One. 2022 Oct 27;17(10):e0274578. doi: 10.1371/journal.pone.0274578 (PMC9612475; doi:10.1371/journal.pone.0274578)

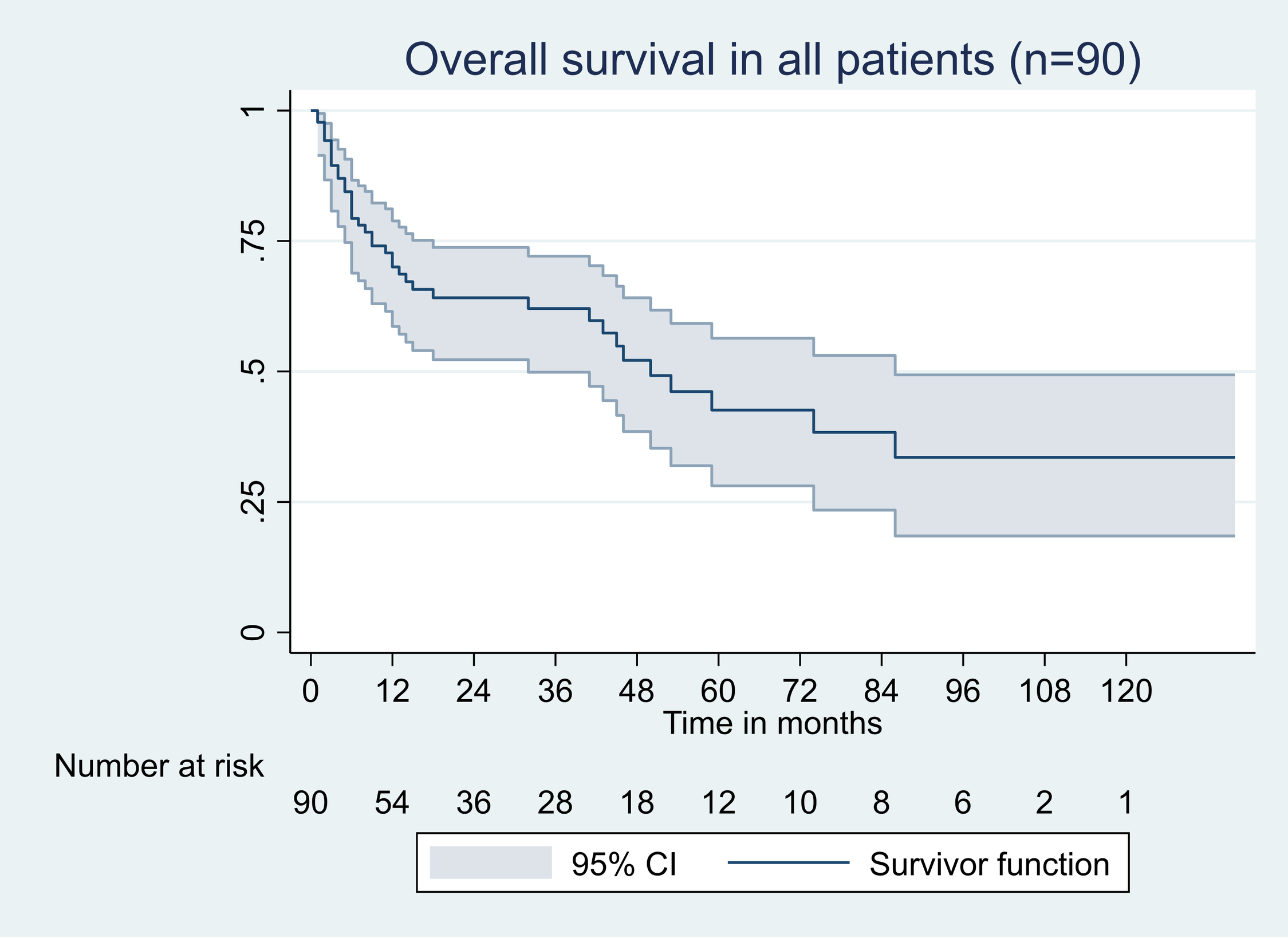

Supplement: S1 Fig — CI, confidence intervals. (TIF) [file pone.0274578.s001.tif]
